# Supplementary material for: Reference Intervals for Platelet Counts in the Elderly: Results from the Prospective SENIORLAB Study
Source: J Clin Med. 2020 Sep 3;9(9):2856. doi: 10.3390/jcm9092856 (PMC7564319; doi:10.3390/jcm9092856)
Supplement: Supplementary file 1 [file jcm-09-02856-s001.pdf]

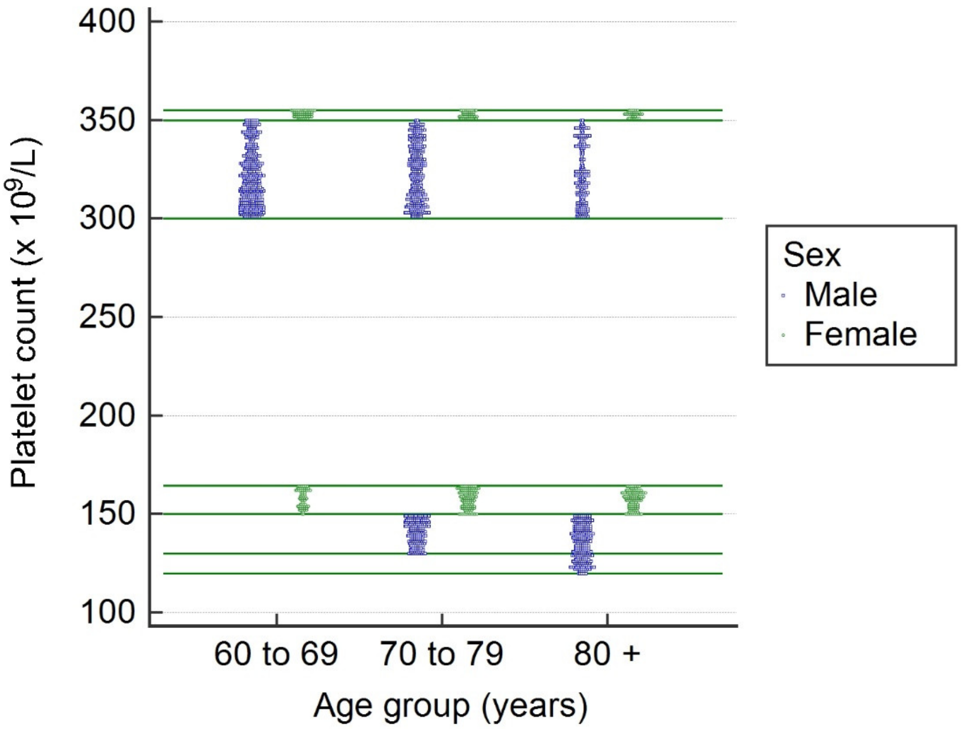

Supplemental Figure 1. Platelet counts measured in Liechtenstein from 1 January 2013 to 31 December 2019, stratified by age and sex: results with different interpretation according to the employed reference interval. Cases that are below (in males) and above (in females) the conventional as well as above (in males) and below (in females) the age- and sex-stratified reference intervals are shown. Green lines represent conventionally used sex- and age-independent reference interval (150–350 × 109/L) [21] as well as the sex- and age-dependent reference intervals evaluated in the SENIORLAB cohort (i.e. 165–355 × 109/L in females; 150–300 × 109/L in males aged 60-69 years, 130–300 × 109/L in males aged 70-79 years, and 120–300 × 109/L in males aged 80 years and more).
